# Sensitive Assessment of the Virologic Outcomes of Stopping and Restarting Non-Nucleoside Reverse Transcriptase Inhibitor-Based Antiretroviral Therapy

**DOI:** 10.1371/journal.pone.0069266

**Published:** 2013-07-18

**Authors:** Anna Maria Geretti, Zoe Fox, Jeffrey A. Johnson, Clare Booth, Jonathan Lipscomb, Lieven J. Stuyver, Gilda Tachedjian, John Baxter, Giota Touloumi, Clara Lehmann, Andrew Owen, Andrew Phillips, for the INSIGHT Strategies for Management of Antiretroviral Therapy (SMART) Study Group

**Affiliations:** 1 Institute of Infection & Global Health, University of Liverpool, Liverpool, United Kingdom; 2 Institute of Neurology, University College London, London, United Kingdom; 3 Division of HIV/AIDS Prevention, Centers for Disease Control, Atlanta, Georgia, United States of America; 4 Department of Virology, Royal Free London NHS Foundation Trust, London, United Kingdom; 5 Janssen Diagnostics BVBA, Beerse, Belgium; 6 Tachedjian Laboratory, Burnet Institute, Melbourne, Australia; 7 Department of Infectious Diseases, Cooper University Hospital, Camden, New Jersey, United States of America; 8 Department of Epidemiology and Statistics, Athens University Medical School, Athens, Greece; 9 Department of Internal Medicine, University of Cologne, Cologne, Germany; 10 Department of Molecular and Clinical Pharmacology, University of Liverpool, Liverpool, United Kingdom; 11 Institute of Epidemiology & Health, University College London, London, United Kingdom; University of Pittsburgh, United States of America

## Abstract

**Background:**

Non-nucleoside reverse transcriptase inhibitor (NNRTI)-resistant mutants have been shown to emerge after interruption of suppressive NNRTI-based antiretroviral therapy (ART) using routine testing. The aim of this study was to quantify the risk of resistance by sensitive testing and correlate the detection of resistance with NNRTI concentrations after treatment interruption and virologic responses after treatment resumption.

**Methods:**

Resistance-associated mutations (RAMs) and NNRTI concentrations were studied in plasma from 132 patients who interrupted suppressive ART within SMART. RAMs were detected by Sanger sequencing, allele-specific PCR, and ultra-deep sequencing. NNRTI concentrations were measured by sensitive high-performance liquid chromatography.

**Results:**

Four weeks after NNRTI interruption, 19/31 (61.3%) and 34/39 (87.2%) patients showed measurable nevirapine (>0.25 ng/ml) or efavirenz (>5 ng/ml) concentrations, respectively. Median eight weeks after interruption, 22/131 (16.8%) patients showed ≥1 NNRTI-RAM, including eight patients with NNRTI-RAMs detected only by sensitive testing. The adjusted odds ratio (OR) of NNRTI-RAM detection was 7.62 (95% confidence interval [CI] 1.52, 38.30; p = 0.01) with nevirapine or efavirenz concentrations above vs. below the median measured in the study population. Staggered interruption, whereby nucleos(t)ide reverse transcriptase inhibitors (NRTIs) were continued for median nine days after NNRTI interruption, did not prevent NNRTI-RAMs, but increased detection of NRTI-RAMs (OR 4.25; 95% CI 1.02, 17.77; p = 0.03). After restarting NNRTI-based ART (n = 90), virologic suppression rates <400 copies/ml were 8/13 (61.5%) with NNRTI-RAMs, 7/11 (63.6%) with NRTI-RAMs only, and 51/59 (86.4%) without RAMs. The ORs of re-suppression were 0.18 (95% CI 0.03, 0.89) and 0.17 (95% CI 0.03, 1.15) for patients with NNRTI-RAMs or NRTI-RAMs only respectively vs. those without RAMs (p = 0.04).

**Conclusions:**

Detection of resistant mutants in the rebound viremia after interruption of efavirenz- or nevirapine-based ART affects outcomes once these drugs are restarted. Further studies are needed to determine RAM persistence in untreated patients and impact on newer NNRTIs.

## Introduction

The SMART trial randomized HIV-1 infected patients with CD4 counts >350 cells/mm^3^ to take antiretroviral therapy (ART) either continuously or episodically, guided by the CD4 cell count [1]. Results showed that interrupting treatment carried a significant risk of morbidity and mortality. There remain circumstances when ART discontinuation may be required (e.g., due to toxicity), or may occur unplanned due to patient choice or problems with drug supply (e.g., in resource-limited settings). In patients receiving ART with agents that have different elimination half-lives, simultaneous interruption of all drugs can lead to a period of inadvertent monotherapy, which can result in viral replication in the presence of a single drug, promoting selection of drug-resistant mutants. This is expected to be a problem especially with the non-nucleoside reverse transcriptase inhibitors (NNRTIs), as they show the longest plasma half-lives among available antiretrovirals [2]. NNRTI clearance rates show significant inter-person variability, however, reflecting the activity of enzymes responsible for NNRTI metabolism, which in turn are influenced by multiple encoding and regulatory genes [3], [4]. A low genetic barrier to resistance further compounds the problem of stopping NNRTI-based ART, as a single mutation in reverse transcriptase (RT) is typically sufficient to abrogate drug activity [5]. It can therefore be proposed that selection of NNRTI resistance may occur in patients stopping NNRTI-based ART and that the risk is higher the slower the NNRTI clearance rate. However, previous studies investigating the correlation between NNRTI concentrations after treatment interruption and detection of NNRTI resistance have not been conclusive, possibly due to small numbers and low sensitivity of testing methods [6], [7]. The extent to which treatment interruption leads to emergence of drug-resistant virus is important for understanding the full implications of stopping ART in relation to both subsequent treatment outcomes and risk of transmission of drug-resistant HIV.

The risk of resistance after interruption of NNRTI-based ART has been previously estimated using Sanger sequencing [6]–[9]. We reported that among 141 patients who interrupted NNRTI-based ART within SMART, 18 (13%) had evidence of NNRTI resistance in the two months following interruption [8]. Sanger sequencing fails to detect mutants present in the viral quasispecies at a frequency below approximately 20%, suggesting that an even greater proportion of patients may carry resistant mutants below this detection limit. The issue is especially relevant to NNRTI therapy. Low-frequency NNRTI-resistant mutants have been detected in both ART-naive and NNRTI-experienced patients with and without high-frequency mutants, and shown to impair responses to NNRTI-based ART [10], [11]. Recommended strategies to minimize the potential risk of drug resistance after interruption of NNRTI-based ART include stopping the NNRTI first and continuing the remaining drugs in the regimen for a short period, commonly the nucleos(t)ide RT inhibitors (NRTIs) (staggered interruption), or replacing the NNRTI with a ritonavir-boosted protease inhibitor (PI/r) for a short period (switched interruption) [12]. There is limited evidence supporting one particular strategy. In a previous study using Sanger sequencing, no NNRTI-RAMs were detected in virologically suppressed children that stopped nevirapine or efavirenz according to either a staggered or a switched interruption modality [9]. Within SMART, we previously reported that both the detection of drug resistance after interruption (by Sanger sequencing) and re-suppression rates after restarting therapy were higher among patients with staggered or switched interruption relative to those with simultaneous interruption [8]. Expanding our previous observations, the aim of this study was to obtain a more accurate estimation of the risk of NNRTI resistance after interruption of NNRTI-based ART by sensitive testing with allele-specific (AS)-PCR and ultra-deep sequencing (UDS). We then investigated the correlation between detection of NNRTI resistance and NNRTI concentrations after treatment interruption, and analyzed the findings in relation to virologic responses after resumption of NNRTI-based ART.

## Methods

### Study Population

Eligible patients were receiving NNRTI-based ART, had a plasma HIV-1 RNA load (‘viral load’) <400 copies/ml, and were randomized to the drug conservation arm of SMART and thus to undergo a treatment interruption [1]. A total of 132/984 (13.4%) patients who interrupted suppressive NNRTI-based ART in SMART and had stored plasma samples available for testing were included in this sub-study. The modality of interruption was chosen by the treating physician, as previously described [8]. Therapy was re-started when the CD4 count decreased <250 cells/mm^3^ or at the occurrence of clinical events [1].

### Ethics Statement

The Institutional Review Board at the University of Minnesota approved the proposal for the use of stored specimens. All necessary permits were obtained for the described study, which complied with all relevant regulations. The samples used in this study have been described in previous publications (references 1 and 8).

### Drug Concentrations

Efavirenz and nevirapine concentrations were measured by validated [13], [14], highly sensitive high-performance liquid chromatography (HPLC) in plasma samples collected at week 4 (visit 1) after NNRTI interruption. The assay lower limit of quantification was 0.25 ng/ml for nevirapine and 5 ng/ml for efavirenz.

### Drug Resistance

Plasma samples collected 4–12 weeks after NNRTI interruption were used for resistance testing. Selection was based upon sample availability and viral load levels >3000 copies/ml to allow reliable testing by the sensitive assays. Samples underwent Sanger sequencing and AS-PCR as previously described [15]–[17]. The AS-PCR targeted the NNRTI resistance-associated mutations (RAMs) K103N, Y181C, and Y188L; samples showing K103N were also tested for G190A. In addition samples were screened for the presence of NRTI-RAMs, including thymidine analogue mutations (TAMs), K65R, Q151M and M184V/I. Mutation-specific interpretative cut-offs ranging from 0.3% to 1% were applied as previously described [15]. In a subset of 21 samples, UDS of the RT amino acid region 100 to 190 (_RT100–190_UDS) was performed as previously described [18]; samples were selected randomly from three subsets according to volume availability: samples with RAMs by AS-PCR; samples without RAMs by AS-PCR; and samples that failed the AS-PCR reaction. Briefly, viral RNA was extracted from 500 µl of plasma (EasyMag, Biomérieux, France) and reverse transcribed into cDNA using the Accuscript HF RT enzyme (Agilent, Santa Clara, USA) and random hexamers. The RT region spanning amino acids 100 to 190 was amplified by nested PCR, and pooled barcoded amplicons were sequenced on the GS-FLX instrument (454 Life Sciences, Roche, Branford, USA) according to the manufacturer’s standard protocol. The experiment was designed to reach on average a mutation detection sensitivity of 1% and an average coverage of 5,500 reads per position was obtained. Amplicons were sequenced from both ends (forward and reverse). The Amplicon Variant Analyzer (AVA) software (Roche) was used for read mapping and calculating variant frequencies at each nucleotide position relative to HIV-1 reference strain HXB2. The presence of relevant mutations was manually verified by inspection of the individual ﬂowgrams. A detection limit of 1% was chosen to avoid the high probability of technical artifacts below this threshold [19]. Major RAMs were assigned according to the International IAS-USA list (Nov 2011).

### Statistical Analysis

Factors associated with the detection of RAMs by all testing modalities combined (n = 131) were investigated using standard univariable and multivariable logistic regression analysis. All factors of interest were stipulated *a priori* and included in the multivariable models. In the first model, the variables analyzed were age, gender, ethnicity, HIV-1 transmission risk group, nadir CD4 count, duration of ART before interruption, viral load and CD4 count at the time of interruption, and interruption modality. In a second model exploring factors associated with the detection of NNRTI-RAMs, the analysis also included nevirapine and efavirenz plasma concentrations (n = 70). To overcome the limitation related to the small number of observations, the drug concentration data were pooled and analyzed as categorical variables (either above or below the median concentration measured in the study population). This approach was stipulated *a priori* as there was insufficient statistical power to analyze the two drugs separately or assess the interaction between nevirapine and efavirenz in this model. The proportion of patients who regained virologic suppression <400 copies/ml after re-starting NNRTI-based ART was investigated using logistic regression analysis as an intention-to-treat switch = failure analysis. Only patients restarting ART without a PI and with at least one viral load measurement in the following 4–12 months were included (n = 90). All factors of interest were stipulated *a priori* and included in the multivariable models. The variable included were age, gender, duration of ART before interruption, viral load and CD4 count at the time of interruption, time between interrupting and restarting ART, the NNRTI restarted, the interruption modality and the presence of RAMs. P-values were not corrected for multiple comparisons. All statistical analyses were performed using STATA software (StataCorp. 2007. Stata Statistical Software: Version 10.2/SE, College Station, Texas, USA).

## Results

### Study Population

The analysis included 132 patients that interrupted efavirenz (n = 80, 60.6%), nevirapine (n = 51, 38.6%), or delavirdine (n = 1, 0.8%) in SMART. All patients were also receiving ≥1 NRTI, comprising lamivudine (n = 86, 65.1%), tenofovir (n = 55; 41.7%), zidovudine (n = 48, 36.4%), emtricitabine (n = 22, 16.7%), abacavir (n = 20; 15.1%), didanosine (n = 19; 14.4%), or stavudine (n = 16, 12.1%). In addition, 15/132 (11.4%) patients were receiving a PI. The modality of interruption was simultaneous in 63/132 (47.7%) patients, staggered in 46/132 (34.8%) patients, and switched in 23/132 (17.4%) patients. The latter two groups continued the NRTIs or replaced the NNRTI with a PI/r respectively for median nine days (interquartile range, IQR 7, 14) before stopping all therapy. The characteristics of the study population at the time of NNRTI interruption and according to the interruption modality are summarized in Table 1. At the time of interruption all patients showed a viral load <400 copies/ml. Overall 113/132 (85.6%) patients had an “undetectable” viral load, but the lower limit of quantification varied by site and was <400 copies/ml in 30/132 (22.7%) patients, <75 copies/ml in 16/132 (12.1%) patients, and <50 copies/ml in 67/132 (50.8%) patients. A further 19/132 (14.4%) patients had a viral load between 50 and 400 copies/ml (median 175 copies/ml; range 55 to 394 copies/ml).

**Table 1:** Characteristics of the study population that interrupted NNRTI-based ART in SMART, according to the interruption modality.^a^

			Total	Interruption modality		
				Simultaneous	Staggered	Switched
			n = 132	n = 63	n = 46	n = 23
Male gender, n (%)			99 (75.0)	47 (74.6)	32 (69.6)	20 (87.0)
Risk group, n (%)	MSM		61 (46.2)	31 (49.2)	18 (39.1)	12 (57.2)
	Heterosexual		41 (31.1)	20 (31.8)	18 (39.1)	3 (13.0)
	Other/Unknown		30 (22.7)	12 (19.0)	10 (21.7)	8 (34.8)
Ethnicity, n (%)	Black		51 (38.6)	22 (34.9)	21 (45.7)	8 (34.8)
	White		63 (47.7)	34 (54.0)	18 (39.1)	11 (47.8)
	Other/unknown		18 (13.6)	7 (11.1)	7 (15.2)	4 (17.4)
CDC category C, n (%)			31 (23.5)	11 (17.5)	13 (28.3)	7 (30.4)
HIV-1 RNA load <50 copies/ml, n (%)^b^			67 (50.8)	26 (41.3)	29 (63.0)	12 (52.2)
Age, median years (IQR)			45 (39, 52)	44 (39, 50)	48 (41, 52)	45 (41, 54)
CD4 count, median cells/mm^3^ (IQR)			645 (475, 793)	624 (475, 833)	657 (461, 758)	643 (527, 751)
Nadir CD4 count, median cells/mm^3^ (IQR)			207 (90, 308)	212 (115, 374)	199 (67, 303)	205 (70, 300)
Time on ART, median years (IQR)			6 (3, 9)	6 (3, 9)	7 (3, 9)	6 (3, 10)

a Patients interrupted ART by simultaneously interrupting all drugs, continuing the nucleos(t)ide reverse transcriptase inhibitors (NRTIs) for a short period, or switching to a ritonavir-boosted protease inhibitor for a short period, referred to as simultaneous, staggered and switched interruption respectively.

b In 46 patients the viral load was measured by assays with a lower limit of quantification of either 75 or 400 copies/ml and results were “undetectable” below these cut-offs; 19 patients showed a viral load between 50 and 400 copies/ml. NNRTI = non-nucleoside reverse transcriptase inhibitor; ART = antiretroviral therapy; MSM = Men who have sex with men; IQR = interquartile range.

### Drug Concentrations

NNRTI concentrations were measured in 70 patients with sufficient plasma samples available at week 4, the first study visit after NNRTI interruption. Median 32 days (IQR 27, 38) after interruption, nevirapine was detected in 19/31 (61.3%) patients at a median concentration of 2.2 ng/ml (IQR 1.0, 49). Median 30 days (IQR 27, 34) after interruption, efavirenz was detected in 34/39 (87.2%) patients at a median concentration of 21 ng/ml (IQR 11, 61). Overall median concentrations (calculated by assigning a value of zero to results below the assay cut-off) were 1.0 ng/ml for nevirapine (n = 31) and 16 ng/ml for efavirenz (n = 39).

### Drug Resistance

Resistance testing was performed in samples collected median 8 weeks (IQR 4, 11) after NNRTI discontinuation. At the time of testing, the median viral load was 27,618 copies/ml (IQR 8,480, 76,200). Resistance results by were obtained in 131/132 (99.2%) samples, comprising 122 Sanger sequencing results, 124 AS-PCR results, and 21 _RT100–190_UDS results (Table 2). Overall, 116 samples had results by both Sanger sequencing and sensitive resistance testing. Six samples failed the AS-PCR reaction and had only Sanger sequencing results (including one sample with NRTI-RAMs); two further samples failed the AS-PCR reaction and had both Sanger sequencing results (including one sample with NRTI-RAMs) and _RT100–190_UDS results (no RAMs detected). Nine samples did not have sufficient volume and were tested only by AS-PCR (no RAMs detected). One sample failed to give a result by both Sanger sequencing and AS-PCR (_RT100–190_UDS not done). Of the 21 samples tested by both AS-PCR and _RT100–190_UDS, 21 yielded a result by _RT100–190_UDS and 19 yielded a result by AS-PCR.

**Table 2:** Summary of resistance results obtained by all methodologies.^a^

Method	Number
SS+AS-PCR	95
SS+AS-PCR+UDS	19
SS+UDS	2
SS only	6
AS-PCR only	9
None	1
Total	132

a Plasma samples underwent testing by Sanger sequencing (SS), allele-specific PCR (AS-PCR) and ultra-deep sequencing (UDS) targeting mutations in HIV-1 reverse transcriptase.

The prevalence of ≥1 NNRTI-RAM by all testing modalities was 22/131 (16.8%) overall. Sensitive testing detected ≥1 NNRTI-RAM in 8/116 (6.9%) samples lacking NNRTI-RAMs by Sanger sequencing, and also increased the number of NNRTI-RAMs detected in each sample (Table 3). With 19 samples that underwent both tests, _RT100–190_UDS confirmed the AS-PCR results, with the exception of one sample that showed G190A by _RT100–190_UDS but not by AS-PCR; the frequency of the G190A mutant was 4% by _RT100–190_UDS. In addition _RT100–190_UDS detected NNRTI-RAMs not targeted by AS-PCR, including V179D, L100I, and K101E. Detection of NNRTI-RAMs according to the interruption modality was 13/62 (21.0%) for simultaneous interruption, 8/46 (17.4%) for staggered interruption, and 1/23 (4.3%) for switched interruption.

**Table 3:** Resistance-associated mutations in reverse transcriptase at week 8 after interruption of NNRTI-based ART, according to the interruption modality, HIV-1 RNA load at interruption, and NNRTI concentrations at week 4 post-interruption.

Interruption modality	Interrupted ART regimen	Viral load (copies/ml)	NNRTI concentration (ng/ml)	Resistance-associated mutations^a^
Simultaneous	ZDV/3TC NVP	216	3.2	NNRTI: G190A
	ZDV/3TC NVP	<400	<0.25	NNRTI: Y181C
	ZDV/3TC NVP	<50	1.0	NNRTI: K103N
	TDF/FTC NVP	<50	1.3	NNRTI: **K103N**
	TDF/FTC EFV	<50	16	NNRTI: **K103N**
	TDF/FTC EFV	<75	64	NNRTI: Y188C
	TDF 3TC NVP	<75	NA	NNRTI: **K103N** (21) **Y181C** (6) **G190A** (4)
	TDF 3TC NVP	<50	NA	NNRTI: K101E **Y181C** G190S
	D4T 3TC EFV	<75	41	NNRTI: **V179D** (10) Y188L (19); NRTI: M184V (15)
	ABC 3TC EFV	<400	NA	NNRTI: K103N; NRTI: L74V
	TDF ZDV NVP	<50	NA	NNRTI: **K103N** G190S
	DDI NVP IDV	<400	>250	NNRTI: Y181C; NRTI: M41L D67E T69ins M184V L210W T215Y
	TDF DDI EFV	357	NA	NNRTI: **L100I** (6) **K101E** (2) K103N (98) **G190A** (14); NRTI: **K65R**
Staggered	ZDV/3TC NVP	<400	49	NNRTI: **L100I** (3) **K101E** (8)**;** NRTI : **M41L D67N M184V** (3) L210W T215Y
	ZDV/3TC NVP	<50	1.6	NNRTI: **Y181C;** NRTI: **M184V K219Q**
	ZDV/3TC EFV	<400	15	NNRTI: A98G **K103N**
	ZDV/3TC EFV	<50	<5	NNRTI: **K103N** (5) **V179D** (10)**;** NRTI: **K70R**
	DDI D4T NVP	<400	NA	NNRTI: **Y181C**
	DDI D4T EFV	238	67	NNRTI: K103N **Y181C Y188L G190A; NRTI:** Q151M **M184I** **T215F**
	DDI NVP NFV	375	NA	NNRTI: G190A
	TDF EFV LPV/r	<75	NA	NNRTI: G190EQR; NRTI: D67N T69A K70R T215F K219Q
Switched	ZDV/3TC NVP	<50	0.6	NNRTI: **K103N**

Each row gives data for a single patient.

a Resistance-associated mutations (RAMs) in reverse transcriptase (RT) were detected by Sanger sequencing, allele-specific PCR (AS-PCR) and ultra-deep sequencing (UDS). AS-PCR targeted the NNRTI-RAMs K103N, Y181C, Y188L and G190A (the latter only in samples with K103N), and the following NRTI-RAMs: thymidine analogue mutations (M41L, D67N, K70R, L210W, T215Y/F, K219Q), K65R, Q151M, and 184V/I. Mutation-specific cut-offs ranging between 0.3% and 1% were used for AS-PCR interpretation as previously described [15]. UDS targeted the RT amino acid region 100–190; a detection limit of 1% was chosen to avoid the high probability of technical artifacts below this threshold [19]. RAMs detected by sensitive testing but not by Sanger sequencing are indicated in bold; RAMs detected by _RT100–190_UDS but not by AS-PCR are underlined; where _RT100–190_UDS results were obtained the frequency (%) of the mutant is given in brackets. Only samples with NNRTI-RAMs are shown; an additional 15 samples had NRTI-RAMs only, without NNRTI-RAMs. NNRTI = non-nucleoside reverse transcriptase inhibitor; ART = antiretroviral therapy; ZDV = zidovudine; 3TC = lamivudine; NVP = nevirapine; TDF = tenofovir; FTC = emtricitabine; EFV = efavirenz; D4T = stavudine; ABC = abacavir; DDI = didanosine; IDV = indinavir; NFV = nelfinavir; LPV/r = lopinavir/ritonavir; NA = not available.

The prevalence of ≥1 NRTI-RAM by all testing modalities was 24/131 (18.3%) overall. The NRTI-RAMs comprised TAMs in 20/24 patients, with mean 2.4 TAMs per patient; M184I/V in 15/24 patients; K65R in 3/25 patients; and L74V in 1/25 patient. Sensitive testing detected ≥1 NRTI-RAM in 2/116 (1.6%) patients lacking NRTI-RAMs by Sanger sequencing. These included K65R after simultaneous interruption of tenofovir, didanosine, and efavirenz (viral load at interruption 357 copies/ml), and K70R after staggered interruption of zidovudine/lamivudine and efavirenz (viral load at interruption <50 copies/ml) (Table 3). Sensitive testing also detected additional NRTI-RAMs in two samples already showing NRTI-RAMs by Sanger sequencing (Table 3). With 19 samples that underwent both tests, _RT100–190_UDS confirmed the AS-PCR results, with the exception of one sample that showed M184V by _RT100–190_UDS but not by AS-PCR; the frequency of the M184V mutant was 3% by _RT100–190_UDS. Detection of NRTI-RAMs according to the interruption modality was 10/62 (16.1%) for simultaneous interruption, 13/46 (28.3%) for staggered interruption, and 1/23 (4.3%) for switched interruption.

In total, 13/131 (9.9%), 9/131 (6.9%) and 15/131 (11.4%) patients had ≥1 NNRTI-RAM, both NRTI-RAMs and NNRTI-RAMs, and NRTI-RAMs only, respectively.

### Predictors of Drug Resistance

Detection of NNRTI-RAMs was less likely in patients with a viral load recorded as <50 copies/ml at the time of treatment interruption with an adjusted odds ratio (OR) of 0.28 (95% confidence interval, CI 0.09, 0.91; p = 0.03) (Table 4). There was also a trend towards a reduced risk of NNRTI-RAMs after a switched interruption relative to a simultaneous interruption. NNRTI-RAMs were detected in 10/31 (32.3%) patients with week 4 NNRTI concentrations above the median level measured in the study population (1 ng/ml for nevirapine and 16 ng/ml for efavirenz), and 2/34 (5.9%) in patients with concentrations below the median level (p = 0.007). A separate multivariable model was used to assess the association between NNRTI-RAMs and drug concentrations, to account for the fact that drug concentrations were only available in 70 patients. In this analysis, NNRTI-RAM detection was more likely in patients with NNRTI concentrations above the median levels (>1 ng/ml for nevirapine and >16 ng/ml for efavirenz) with an adjusted OR of 7.62 (95% CI 1.52, 38.30; p = 0.01). Detection of NRTI-RAMs was associated with duration of ART exposure prior to interruption with an adjusted OR of 1.26 for each year longer (95% CI 1.10, 1.45; p = 0.001); the nadir CD4 count with an adjusted OR 0.68 for each 50 cells/mm^3^ higher (95% CI 0.52, 0.87; p = 0.003); and staggered interruption relative to simultaneous interruption with an adjusted OR of 4.25 (95% CI 1.02, 17.77; p = 0.03).

**Table 4:** Predictors of detection of NNRTI resistance-associated mutations (RAMs) after interruption of NNRTI-based ART.^a^

		Univariable analysis			Multivariable analysis		
		OR	95% CI	P-value	OR	95% CI	P-value
Gender	Female	1.14	0.38, 3.49	0.81	0.83	0.16, 4.31	0.83
	Male	1.00	–		1.00	–	
Age	Each 5 years older	1.00	0.78, 1.30	0.97	0.99	0.72, 1.37	0.95
Ethnicity	White	0.48	0.17, 1.30	0.13	0.41	0.11, 1.46	0.20
	Other/Unknown	0.19	0.02, 1.63		0.18	0.02, 1.75	
	Black	1.00	–		1.00		
Risk group	Heterosexual	0.94	0.29, 3.07	0.78	0.54	0.10, 2.85	0.48
	Other/Unknown	0.68	0.08, 6.12		0.43	0.04, 4.52	
	MSM	1.00	–				
Nadir CD4 count	Each 50 cells/mm^3^ higher	0.94	0.81, 1.10	0.47	1.01	0.83, 1.23	0.91
Time on ART pre-interruption	Each year longer	1.03	0.93, 1.15	0.54	1.05	0.91, 1.20	0.49
HIV-1 RNA load at interruption^b^	<50 copies/ml	0.34	0.12, 0.95	0.04	0.28	0.09, 0.91	0.03
	Other	1.00	–		1.00	–	
CD4 count at interruption	Each 50 cells/mm^3^ higher	0.94	0.83, 1.05	0.28	0.93	0.81, 1.08	0.35
Interruption modality	Staggered	0.71	0.25, 1.98	0.17	0.80	0.23, 2.74	0.20
	Switched	0.19	0.02, 1.54		0.11	0.01, 1.10	0.17
	Simultaneous	1.00	–		1.00	–	

a Detection of NNRTI-RAMs by all testing modalities. NNRTI concentrations were analyzed in a separate model due to smaller numbers.

b As noted above some patients had the viral load measured by assays with a lower limit of quantification of either 75 or 400 copies/ml. NNRTI = non-nucleoside reverse transcriptase inhibitor; ART = antiretroviral therapy; OR = Odds ratio; CI = confidence interval; MSM = Men who have sex with men.

### Virologic Responses after Restarting NNRTI-based ART

Patients restarted ART median 29 weeks (IQR 13, 65) after interruption. The analysis of responses was restricted to 90 patients who restarted NNRTI-based ART (58 with efavirenz and 32 with nevirapine) without a PI and had at least one viral load measurement while on the NNRTI-based regimen in the 4–12 months after restarting. Overall, 73/90 (81.1%) patients regained virologic suppression <400 copies/ml; 50/90 (55.5%) had a viral load recorded as <50 copies/ml. The proportion of patients with viral load <400 copies/ml was 8/13 (61.5%) with NNRTI-RAMs, 7/11 (63.6%) with NRTI-RAMs only, and 51/59 (86.4%) without RAMs (p = 0.04); and 32/42 (76.2%), 23/29 (79.3%), and 18/19 (94.7%) following simultaneous, staggered, and switched interruption, respectively (p = 0.18). In multivariable analyses (Table 5), the adjusted OR of suppression <400 copies/ml was 0.18 (95% CI 0.03, 0.89) for patients with NNRTI-RAMs and 0.17 (95% CI 0.03, 1.15) for patients with NRTI-RAMs only, relative to patients without RAMs (p = 0.04). The impact of the interruption modality on likelihood of suppression <400 copies/ml became more evident 12–18 months after restarting ART, when the multivariable model showed an adjusted OR of suppression <400 copies/ml of 10.01 for staggered/switched interruption relative to simultaneous interruption (95% CI 1.73, 58.34; p = 0.01) (data not shown).

**Table 5:** Predictors of virologic suppression <400 copies/ml 4–12 months after restarting NNRTI-based ART.^a^

		Univariable analysis			Multivariable analysis		
		OR	95% CI	P-value	OR	95% CI	P-value
Gender	Female	0.60	0.19, 1.85	0.38	0.41	0.10, 1.68	0.22
	Male	1.00	–		1.00	–	
Age	Each 5 years older	1.04	0.78, 1.39	0.79	1.09	0.71, 1.68	0.70
Time on ART pre-interruption	Each year longer	0.91	0.80, 1.03	0.15	0.89	0.75, 1.07	0.21
HIV-1 RNA load at interruption^b^	<50 copies/ml	1.09	0.38, 3.15	0.87	1.00	0.27, 3.72	0.99
	Other	1.00	–		1.00	–	
CD4 count at interruption	Each 50 cells/mm^3^ higher	1.05	0.91, 1.19	0.52	1.06	0.89, 1.27	0.50
Time to restarting ART	Each week longer	1.01	0.99, 1.04	0.21	1.02	0.99, 1.06	0.11
NNRTI restarted	Nevirapine	0.55	0.19, 1.61	0.28	0.55	0.15, 2.09	0.38
	Efavirenz	1.00	–		1.00	–	
Interruption modality	Staggered/Switched	1.83	0.63, 5.34	0.27	2.62	0.60, 11.38	0.20
	Simultaneous	1.00	–		2.62	–	0.20
Resistance-associated mutations	NNRTI	0.22	0.06, 0.84	0.03	0.18	0.03, 0.89	0.04
	NRTI only	0.24	0.06, 1.01		0.17	0.03, 1.15	
	None	1.00	–		1.00	–	

a The analysis included 90 patients who restarted NNRTI-based ART without a protease inhibitor and had at least one viral load measurement in the 4–12 months after re-starting therapy.

b As noted above some patients had the viral load measured by assays with a lower limit of quantification of either 75 or 400 copies/ml. NNRTI = non-nucleoside reverse transcriptase inhibitor; ART = antiretroviral therapy; OR = Odds ratio; CI = confidence interval.

## Discussion

This study demonstrated RAMs in a substantial number of patients experiencing rebound viremia after stopping suppressive NNRTI-based ART. Interpretation of the findings should take two limitations into consideration. Viral load assays with different lower limits of quantification were used in SMART and viral load suppression <50 copies/ml could not be demonstrated in all patients at study entry. Furthermore, due to sample availability, NNRTI concentrations were obtained only in a subset of patients. Nonetheless, the data provide sufficient evidence to indicate that different factors influenced the detection of RAMs after ART interruption. NNRTI-RAMs were less likely in patients who had a viral load recorded as <50 copies/ml at the time of interruption, indicating a risk of selecting NNRTI resistance even at low levels of viremia between 50 and 400 copies/ml. In addition, NNRTI-RAMs were more likely in patients showing higher plasma NNRTI concentrations at week 4 after interruption. These findings provide support to the notion that selection of NNRTI resistance can occur in patients experiencing slower NNRTI clearance after ART interruption. Two previous studies did not observe an association between NNRTI concentrations after interruption and detection of NNRTI-RAMs [6], [7]. One study found that median efavirenz or nevirapine concentrations at day 15 after interruption did not differ significantly between 12 patients with NNRTI-RAMs (by Sanger sequencing) and 20 patients without RAMs [6]. Of note, the NNRTI was quantifiable in less than a third of available samples. A second study reported that median efavirenz concentrations and rate of efavirenz decline over 7–10 days after interruption did not differ significantly between 7 patients with NNRTI-RAMs (by Sanger sequencing) and 14 patients without RAMs. Thus, both the size of the study population, the timing of the assessment, and the sensitivity of the testing methods for both drug concentrations and NNRTI-RAMs differed in our study compared with the two previous reports. A previous study of 19 patients receiving intermittent efavirenz-based ART assayed efavirenz concentrations and used AS-PCR to detect the NNRTI-RAM K103N during the off-therapy periods. Consistent with our findings, AS-PCR increased detection of NNRTI resistance relative to Sanger sequencing; furthermore the half-life of efavirenz was higher in 8 patients in whom K103N emerged compared with 11 patients in whom it did not (p  =  0.04) [20].

Genetic predictors of NNRTI clearance are being identified which may help tailoring NNRTI discontinuation. The cytochrome P450 (CYP)-2B6 isoenzyme (*CYP2B6*), for instance, catalyzes the main oxidative metabolism reaction for efavirenz. Three polymorphisms within the *CYP2B6* gene have been associated with efavirenz estimated Cmin, although together they explain only one-third of inter-individual variability [4]. Meanwhile, measuring drug levels at week 4 after NNRTI discontinuation may potentially offer a readily available tool to assign patients to the low or high risk of NNRTI-RAMs. It must be pointed out however that the fact that drug concentrations were measured in a subset of patients limits the power of our conclusions. While the dataset was larger than in previous studies, the statistical analysis required a separate model and pooling of the nevirapine and efavirenz concentration data, which involved an underlying assumption that the effect of nevirapine concentrations is the same as that of efavirenz concentrations. The drug concentration data were analyzed as categorical variables either above or below the respective median concentrations measured in the study population. Thus, the results provide proof-of principle evidence that patients with slower nevirapine or efavirenz clearance have a greater risk of NNRTI resistance after interruption, although further studies are required to identify drug-specific cut-offs that are predictive of resistance. Further analyses of interest may also include the correlation between drug concentrations measured before and after treatment interruption. In addition, although we were unable to identify an association between detection of NNRTI-RAMs and the NRTIs used (data not shown), the different half-life of NRTIs has the potential to influence the risk for resistance development [7] and its effects warrant further investigation.

Detection of NRTI-RAMs was surprisingly common in this study. NRTI-RAMs were more likely in patients with a long previous ART history, suggesting re-emergence of resistant mutants archived during previous virologic failures, rather than, or in addition to, *de novo* selection during viral load rebound. In support of this hypothesis, most NRTI-RAMs were detected by Sanger sequencing, and there was a high prevalence of patients showing two or more TAMs. As TAMs are known to emerge in stepwise fashion during prolonged therapy with zidovudine or stavudine [21], it would seem that multiple TAMs were unlikely to arise for the first time solely as a result of treatment interruption. Detailed treatment histories and results of previous resistance tests would be required to corroborate this hypothesis. Interestingly, there was an increased detection of NRTI-RAMs in patients with a staggered interruption, suggesting a potential for selection or re-selection of mutants by the continued NRTIs.

We previously reported that patients who had undergone a simultaneous interruption showed reduced virologic responses after restarting ART compared with those with a staggered or a switched interruption [8]. Here we confirm the previous observation that simultaneous interruption should be avoided when possible. We further propose that a switched interruption may be preferable to a staggered interruption both to offer improved protection against emergence of NNRTI-RAMs and reduce selection of NRTI resistance; the latter may be especially important in patients with previous NRTI experience.

In our previous study we used Sanger sequencing to detect RAMs after ART interruption [8]. Here we demonstrated that sensitive testing increased prevalence and spectrum of NNRTI-RAMs detected during viral load rebound. The data provide a measure of the potential risk of NNRTI resistance after ART interruption. It should be noted that at 16.8%, the overall prevalence of NNRTI-RAMs was lower than that observed in patients either failing NNRTI-based ART or receiving single-dose nevirapine for the prevention of mother to child transmission [10], [22]. This may be explained by the consideration that both sufficient levels of virus replication and sufficient drug concentrations must co-exist to allow selection of drug resistance. The optimal “selection window” is likely to be narrower in patients stopping NNRTI-based ART with a suppressed viral load relative to patients receiving single-dose nevirapine in the presence of a fully replicating virus. A further consideration is that patients interrupting NNRTI-based ART in SMART had already achieved steady-state NNRTI pharmacokinetics through the induction of hepatic enzymes. In addition, testing samples collected several weeks after treatment interruption, while required to ensure adequate viral load levels, may have missed the earlier emergence of resistant strains. Finally, the AS-PCR method applies strict cut-offs for interpreting positivity.

The AS-PCR methodology employed in this study has undergone extensive validation [15]–[17]. In previous studies we demonstrated that low-frequency NNRTI-RAMs detected by AS-PCR were predictive of virologic failure among naive patients starting first-line NNRTI-based ART [11], [16], [17], and also influenced the detection probability and type of NNRTI-RAMs detected at the time of virologic failure [23]. One downside of AS-PCR is that it is mutation-specific and to a large extent clade-specific, and labor-intensive. In recent years, next-generation sequencing methodologies, including UDS, have become available that allow the quantitative detection of mutants with greatly enhanced sensitivity relative to Sanger sequencing (reliably down to a cut-off of about 1%) [19]. Direct comparisons of AS-PCR with UDS are limited. A previous study of 11 samples undergoing AS-PCR for K103N showed a good level of agreement with UDS [20]. Here, using a subset of 21 samples that underwent both AS-PCR and UDS, we found good concordance between the two techniques at the respective validated cut-offs for interpretation. Importantly, although the AS-PCR targeted a relatively small number of NNRTI-RAMs, these were the key RAMs associated with resistance to efavirenz or nevirapine, and the spectrum was only marginally expanded in the samples that also underwent UDS of the RT region spanning amino acids 100 to 190.

In summary, this study provides substantive evidence in support of the widely cited hypothesis that stopping NNRTI-based ART carries a risk of drug resistance. We show that viral load levels at the time of interruption, plasma NNRTI concentrations at week 4 after interruption, overall treatment history, and interruption modality combine to influence the risk of resistance and ultimately predict virologic responses when NNRTI-based ART is resumed. Further studies are required to assess the persistence of NNRTI-RAMs in patients remaining off therapy, the potential for their onward transmission, and the implication of these findings for etravirine and rilpivirine use.
